# A Broad-Spectrum Antimicrobial Activity of *Bacillus subtilis* RLID 12.1

**DOI:** 10.1155/2014/968487

**Published:** 2014-08-11

**Authors:** Ramya Ramachandran, Ajay Ghosh Chalasani, Ram Lal, Utpal Roy

**Affiliations:** ^1^Department of Biological Sciences, BITS Pilani KK Birla Goa Campus, Goa 403726, India; ^2^Department of Microbiology, SBSPGIBMS, Dehradun 248161, India

## Abstract

In the present study, an attempt was made to biochemically characterize the antimicrobial substance from the soil isolate designated as RLID 12.1 and explore its potential applications in biocontrol of drug-resistant pathogens. The antimicrobial potential of the wild-type isolate belonging to the genus* Bacillus *was determined by the cut-well agar assay. The production of antimicrobial compound was recorded maximum at late exponential growth phase. The ultrafiltered concentrate was insensitive to organic solvents, metal salts, surfactants, and proteolytic and nonproteolytic enzymes. The concentrate was highly heat stable and active over a wide range of pH values. Partial purification, zymogram analysis, and TLC were performed to determine the preliminary biochemical nature. The molecular weight of the antimicrobial peptide was determined to be less than 2.5 kDa in 15% SDS-PAGE and in zymogram analysis against* Streptococcus pyogenes*. The N-terminal amino acid sequence by Edman degradation was partially determined to be T-P-P-Q-S-X-L-X-X-G, which shows very insignificant identity to other antimicrobial peptides from bacteria. The minimum inhibitory concentrations of dialysed and partially purified ion exchange fractions were determined against some selected gram-positive and gram-negative bacteria and some pathogenic yeasts. The presence of three important antimicrobial peptide biosynthesis genes* ituc, fend, *and* bmyb *was determined by PCR.

## 1. Introduction

Mechanism of resistance in clinical background reflects very serious problem in the treatment of pathogenic microbes. Serious bacterial and fungal infections are increasingly recognized as important causes of morbidity and mortality, especially among debilitated patients [1, 2]. Hospital-acquired infections are most commonly associated with invasive medical devices or surgical procedures which turn out to be a challenge to patient safety [2]. Famous hospital-acquired infections called “ESKAPE” (*Enterococcus faecium, Staphylococcus aureus, Klebsiella pneumoniae, Acinetobacter* species,* Pseudomonas aeruginosa*, and* Enterobacter* species) are recognized as the most important emerging threats of this century [3].

To confront the growing antimicrobial resistances, modern medicine focus natural products for novel antibiotics and antimicrobials. Strains of* Bacillus subtilis*, the model system for gram-positive organisms, are able to produce more than two dozen antibiotics with different structures and functions depending on the ecological niche or induced systematic resistance [4].* Bacillus* isolates are rather well known for the production of a vast array of structurally unrelated antimicrobial compounds, which include lipopeptides like iturin, surfactin, fengycins, bacteriocins, and bacteriocin like inhibitory substances (BLIS) [5]. The production of lipopeptides allows certain* B. subtilis* strains to modify their outer surface which permits them to regroup together in a biofilm in order to proliferate and spread in the territory. The major types of antimicrobial compounds from* B. subtilis* include peptides that are either ribosomally synthesized and posttranslationally modified (lantibiotics and lantibiotic-like peptides) or nonribosomally generated, as well as a couple of nonpeptidic compounds such as polyketides, an amino sugar, and a phospholipid [5]. The multifarious antimicrobial compounds produced by various* Bacillus* strains have the ability to address the multidrug resistant problems. Only a small portion of the antimicrobial molecules potentially produced by this genus has been identified. From the current investigation we report the preliminary characterization of a novel broad-spectrum antimicrobial substance produced by a soil isolate* B. subtilis* RLID 12.1.

## 2. Materials and Methods

### 2.1. Microbial Strains, Media, and Growth Conditions

The isolate* Bacillus* sp. was grown in Tryptic Soy Broth with additional 0.5% yeast extract (modified Tryptic Soy Broth, pH 7.4) at 37°C under shaking condition. The fungal cultures were maintained in MGYP broths and agar (pH 6.6) at appropriate temperatures and the indicator bacterial strains were grown in Brain Heart Infusion slants (Hi-Media, Bombay, India) at 37°C and maintained at 4°C. The indicator organisms used in this investigation were obtained from Microbial Type Culture Collection (MTCC) Chandigarh, National Collection of Industrial Microorganisms (NCIM) Pune, India, and clinical isolates from hospitals. The cultures were also maintained as 20% glycerol stock at −80°C.

### 2.2. Identification of the* Bacillus* Isolate RLID 12.1

The antibiotic producing* Bacillus* strains RLID 12.1 was initially identified by various biochemical tests like Methyl Red and Voges-Proskauer tests, production of indole and catalase, growth at different temperatures, and different concentrations of NaCl according to Bergey's Manual of Determinative Bacteriology. Carbohydrate fermentation tests were done using KB009 Kit from HiMedia Pdt. Ltd., India.

### 2.3. PCR Based Identification

Total genomic DNA of the selected soil isolate was extracted from 24 h Trypticase Soy Broth cultures. 50 *μ*L reaction volumes PCR was performed using about 100 ng of genomic DNA, 10X reaction buffer, 10 mM (each) deoxynucleoside triphosphates, 1.5 mM MgCl_2,_ and 1.0 U of Taq polymerase (Bangalore GeneI). The universal eubacterial primers 27F (5′-AGA GTT TGA TCM TGG CTC AG-3′) and 1492R (5′-GGT TAC CTT GTT ACG ACT T-3′) [6] at the rate of 100 picomoles were used to amplify small-subunit rRNA (16S rRNA) gene sequences of the wild-type soil isolate.

### 2.4. Inhibitory Spectra

The antimicrobial compound producing* Bacillus* isolate RLID 12.1 was grown in 100 mL modified Tryptic soy broth (mTSB, pH 7.4) at 37°C for 48 h. After 48 h, the supernatant was collected by centrifuging grown culture at 12000 rpm for 30 min and filtered through 0.45 *μ*m polysulphonate membrane (Axiva). Filtered cell-free supernatant was concentrated by ultrafiltration using Millipore membrane (Molecular weight cut-off 3 kDa) in a stirred cell device (Millipore) and was tested using the agar well diffusion assay against selected indicator strains (Table 1). Appropriate agar media plates were swabbed with 100 *μ*L of appropriately diluted freshly grown indicator microorganism and wells of 8 mm diameter were cut and filled with 100 *μ*L of concentrated supernatants [7]. The zone of inhibition was inspected after incubating the plates for 24–48 h at respective temperatures and measured in diameter.

#### 2.4.1. Growth Kinetics

Modified Trypticase Soya broth was inoculated with freshly grown RLID 12.1 and incubated at 37°C. After every 4 h, 2 mL of sample was drawn and centrifuged at 12000 rpm for 30 min and supernatants were membrane-filtered through 0.45 *μ*m disposable filter and assayed for antibacterial activity against* Streptococcus pyogenes* MTCC 442. Simultaneously bacteria growth was recorded spectrophotometrically at OD_600_. The antimicrobial activity was expressed in terms of arbitrary units (AU) which was defined as the highest dilution of the sample that produced a zone of inhibition. The reciprocal of the dilution was considered as the titre of antimicrobial activity (AUml^−1^).

### 2.5. Preliminary Characterization of the Antimicrobial Compound

#### 2.5.1. Effect of Enzymes

To check the sensitivity of enzyme, the cell-free supernatant concentrated by ultrafiltration (using stirred cell and 3 kDa molecular weight cut-off stirred cell membrane, Millipore) was treated with filter-sterilized trypsin (0.5 M Tris HCl buffer, pH 8.0), pronase E (10 mM sodium phosphate buffer, pH 7.0), lipase (100 mM phosphate buffer, pH 7.4), and *α*-amylase (20 mM phosphate buffer, pH 7.0) at a final concentration of 10 mg/mL at 37°C and proteinase K (50 mM Tris HCl buffer, pH 7.5) at a final concentration of 10 mg/mL at 55°C for 2 h. The concentrates without enzyme treatment and buffers were used as positive and negative controls, respectively. The residual activity was determined by agar well diffusion method against* S. pyogenes* MTCC 442 [7].

#### 2.5.2. Stability of the Antimicrobial Activity

To determine thermal stability, aliquots of concentrated supernatants were incubated at different temperatures: 37°C for 5 h, 50°C and 60°C for 3 h, 70°C for 2 h, 80, 90 and 100°C for 1 h, and 121°C for 40 min. pH stability was analyzed for 2 h at 37°C with 0.1 M buffers of different pH: citrate buffer (pH 3.0–5.0), phosphate buffer (pH 6.0 and 7.0), Tris buffer (pH 8.0 and 9.0), and glycine NaOH buffer (pH 10.0) for 2 h. After incubation, antimicrobial activity was checked.

#### 2.5.3. Effect of Surfactants and Other Chemicals

Effect of surfactants like SDS, Tween 20, urea, and Triton X-100 at a final concentration of 1% (v/v), trichloroacetic acid (10 mg/mL (w/v, TCA)), *β*-mercaptoethanol (*β*-ME) at 10% concentration, and EDTA at 2, 5 and 50 mM concentration was analysed by incubating at 37°C for 5 h [8]. Chemicals at the rate of 1% and 10% in mTSB broth and untreated concentrated supernatant were used as negative and positive controls, respectively. After treatment with TCA, samples were centrifuged at 10,000 rpm for 5 min and the supernatant was adjusted to pH 8.0 and checked for antimicrobial activity [9].

#### 2.5.4. Effect of Organic Solvents and Metals

The concentrate was mixed with various organic solvents (ethanol, methanol, isopropanol, acetone, ethyl acetate, chloroform, and isobutanol) at a final concentration of 50% (v/v). After incubation for 1 h at 25°C, the organic solvent was evaporated in a vacuum concentrator (Martin-Christ) and was dissolved in 20 mM Tris HCl buffer pH 8.0. The respective buffers and untreated concentrated supernatant were used as negative and positive controls [9]. Then the residual antimicrobial activity was determined. Effect of metal salts (AgNO_3_, MgSO_4_, MnCl_2_, ZnSO_4_, CuSO_4_, CdCl_2_, FeSO_4_, and CaCl_2_) at a final concentration of 1 mg/mL on concentrated supernatant was analysed by incubating at 37°C for 1 h. The untreated concentrated supernatant and solutions of metals salts were used as positive and negative controls, respectively [8].

#### 2.5.5. Ammonium Sulphate Fractionation

*B. subtilis* RLID 12.1 was grown at 37°C for 48 h. The cell-free supernatant was prepared by centrifuging the growth at 12000 rpm at 4°C. The cell-free supernatant was subjected to sequential ammonium sulphate precipitation to accomplish 30, 50, and 80% saturation at 4°C. Then pellet was resuspended in 20 mM Tris buffer (pH 8.0) and was dialysed using 2 kDa molecular weight cut-off membrane (Spectrapor) against the same buffer for 24 h with four changes at 4°C. The dialyzate was stored at −20°C for further analysis.

### 2.6. Partial Purification of Antimicrobial Compound

The dialysed sample (1 mg/mL) was applied to DEAE Sepharose Fast flow (GE Healthcare) column (110 mm × 17 mm) preequilibrated with 20 mM phosphate buffer (pH 8.0) and protein was eluted with same buffer followed by a gradient from 0–0.5 M at a flow rate of 1 mL/min. Protein in the effluent was monitored by determining the absorbance at 280 nm. A total of 90 fractions (2 mL) were collected, and 100 *μ*L aliquot of each fraction was tested for antibacterial activity by cut-well agar assay. As a negative control for the bioassay the phosphate buffer and broth were used.

### 2.7. N-Terminal Sequencing

To determine the N-terminal amino acid sequence of the sample, the peptide band that showed the activity (in zymogram analysis as shown below) was precisely electroblotted to a 0.45 *μ*m PVDF membrane (Millipore) and then it was subjected to a protein sequencer (model 494, Procise/140C Analyzer, Applied Biosystems, Inc, Iowa State University, USA).The N-terminal sequence of the peptide was determined by using Edman degradation system.

#### 2.7.1. Tricine SDS-PAGE and Zymogram

The biologically active sample was subjected to molecular mass determination by SDS PAGE using Tricine as trailing ions on 15% gel in accordance with the procedure of Laemmli and Favre [11]. Electrophoresis was carried out at 60 V. After electrophoresis, one part of the gel was fixed (25% ethanol, 5% formaldehyde) for 30 min, followed by repeated washing with sterile distilled water for 3 h. To detect proteins bands with antibacterial activity, the gel was washed three times in 0.1% Tween 80 (40 min each wash) at room temperature. Subsequently the SDS-free gel was aseptically placed in Brain Heart Infusion broth (0.7% agar, w/v) containing approximately 10^6^ cells of* S. pyogenes* MTCC 1928 and* S. pyogenes* MTCC 442. The plates were incubated at 37°C for 24 h and observed for the presence of inhibition zone. The other part of the gel containing sample and standard marker (3.5–43 kDa, GeneI) was stained with silver nitrate.

### 2.8. Thin Layer Chromatography (TLC)

Partially purified compound was spotted on silica gel 60 plate (2.0 × 6.0 cm^2^) and was developed with n-butanol-methanol-water (3 : 1 : 1, v/v) as the solvent mixture. The TLC plate was sprayed with water and ninhydrin (0.2%) in order to determine the lipophilicity of the substance and presence of amino acid in the sample, respectively. Also the TLC plate was exposed to iodine vapour to detect the lipid moiety in the compound.

### 2.9. Detection of* iturin C, fengycin*, and* bmy B* Genes

Genomic DNA from strain RLID 12.1 was prepared as described by Neumann et al. [12] and used as a DNA template for the PCR amplifications with specific primers for the* iturin* C,* fenzycin,* and* bmy B* genes listed in Table 2. The PCR mix in a 50 *μ*L reaction volume was as follows: 100 ng of genomic DNA as template, 2.5 U of* Taq* DNA polymerase (EMerck), 5 *μ*L reaction buffer, MgCl_2_ (25 mM), deoxynucleoside triphosphate solution (10 mM each), and of each primer (100 pico moles). The PCR conditions included an initial denaturing step for 2 min at 94°C followed by 30 cycles of denaturing at 94°C for 1 min, annealing at the specific temperature (Table 1) for 45 s, elongation at 72°C for 45 s followed by a final extension of 7 min at 72°C. The PCR products were analyzed by electrophoresis on 1.5% (w/v) agarose gel. Amplification was accomplished in a thermal cycler (MJ Research, Biorad). The gel was run at 80 V for approximately 90 min and 100 bp DNA ladder (Merck) was used as the molecular weight marker.

### 2.10. Determination of Minimum Inhibitory Concentration (MIC)

The MIC for different strains was determined by using a microtiter plate dilution method in appropriate broth using 96-well plates. Using 0.5 McFarland standards, 1 × 10^5^ CFU/mL of each indicator bacteria was prepared and subjected to varying concentration of the dialyzed concentrate of the ammonium sulphate fractionated sample and anion-exchange purified compounds in the range of 64.0–0.125 *μ*g/mL and 200.0–0.39 *μ*g/mL, respectively, by twofold dilution methods [13]. The microtiter plates were incubated at 37°C ± 0.2 incubator under shaking condition. The lowest concentration that inhibited growth of indicator strains was recorded.

## 3. Results

Strain RLID 12.1 was initially identified on the basis of its morphological and biochemical characteristics and according to the results (data not shown here) it belongs to the genus* Bacillus*. Identification was later confirmed by genotypic characterization by PCR amplification, using specific primers [6] and 16S rRNA typing. Based on the 16S rRNA gene sequences, comparative sequences analysis and biochemical characteristics analysis, it can be suggested that the soil-isolate belongs to the genus* Bacillus* with the highest identity to* B. subtilis*. The accession number of RLID 12.1 submitted to the GenBank nucleotide sequence database is JX089317 [14].

### 3.1. Inhibitory Spectrum of RLID 12.1

**Figure 1:**
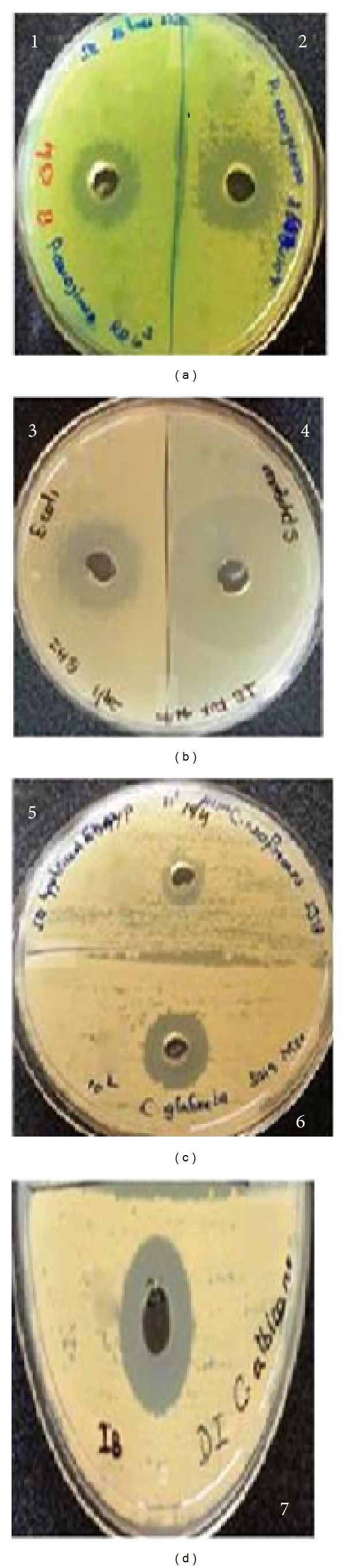
Inhibition zone produced by the test strain* B. subtilis* RLID 12.1. The figures  1, 2, and 3 in Plates (a) and (b) represent antibacterial activity against gram-negative bacteria:* Pseudomonas* sp. (clinical isolate),* P. aeruginosa* MTCC 2582, and* E. coli* MTCC 729, respectively; 4 represents inhibition against gram-positive bacteria,* S. pyogenes* MTCC 442. Figures  5, 6, and 7 in plates (c) and (d) represent antimycotic activity against* C. neoformans* NCIM 3378,* C. glabrata* MTCC 3019, and* C. albicans* DI (clinical isolate, GenBank accession number KJ095700), respectively.

The sensitivity of a wide range of microorganisms to the antimicrobial substance elaborated by strain RLID 12.1 was tested using the cut-well agar assay. The ultrafiltered concentrate (25x, concentration 0.6 mgmL^−1^) of the cell-free supernatant of the strain* B. subtilis* RLID 12.1 exhibited strong and broad-spectrum antimicrobial activity (Figure 1) towards the gram-negative bacteria* Escherichia coli*,* Pseudomonas aeruginosa*,* Klebsiella pneumoniae, Proteus vulgaris, Acinetobacter baumannii,* and* Yersinia aldovae* (Table 2). The antimicrobial activity was observed against the gram-positive bacteria like* Staphylococcus aureus, Streptococcus pyogenes,* and* Enterococcus faecalis* and pathogenic yeasts like* C. albicans, C. glabrata, C. krusei,* and* Cryptococcus neoformans* (Figure 1 and Table 2). The ultrafiltered concentrate was tested against 32 strains and found to be effective against 28 strains examined in this investigation. Results were represented as the mean values with the relevant standard deviation error.

### 3.2. Growth Kinetics

**Figure 2:**
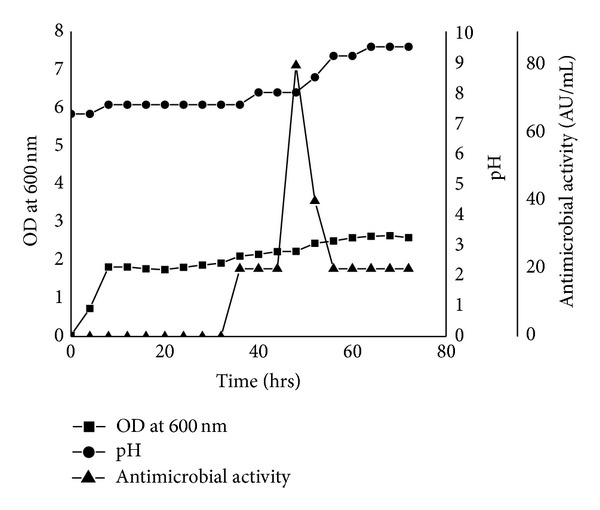
Growth kinetics of the producer strain and antimicrobial substance production expressed in AUml^−1^ (as described in Section 2.4.1); the antibacterial activity of cell-free culture supernatants was assayed by using* S. pyogenes* MTCC 442.

*S. pyogenes* MTCC 442 was used as an indicator organism in studying the production of antimicrobial substance by* B. subtilis* RLID 12.1. The production started at 36 h and attained maximum level at 48 h of growth (Figure 2). Thereafter the antimicrobial activity started dwindling and remained constant till the stationary phase and early decline phase. pH values were checked at every 4 h interval along with the growth and production of antimicrobial substance. It was observed that pH values increased gradually from the initial pH of 7.4 to 9.5 at the death phase. However the pH value was found to be 8.0 during the maximum production (80 AUml^−1^) of the antimicrobial substance (Figure 2).

### 3.3. Effect of Enzymes, pH, Heat, and Chemicals on the Antimicrobial Compound

The characterization studies of antimicrobial compound produced by RLID 12.1 were summarized in Table 3. The antimicrobial peptide was tested for sensitivity to proteolytic and nonproteolytic enzymes, heat, pH, and various chemicals like surfactants, metal salts, and solvents. There was no loss of antimicrobial activity upon exposure to pronase E, trypsin, *α*-amylase, and lipase at a concentration of 10 mgml^−1^. However the activity was retained up to 72% when treated by proteinase K at the concentration of 10 mgml^−1^. The respective buffers used as controls showed no activity.

The antimicrobial activity was recorded over a wide range of pH (3.0−10.0); 100% activity was found between pH 6.0 and 8.0. The antimicrobial substance was found to be heat stable at all temperatures tested. 100% activity was retained at 37°C for 5 h, 95% and 88% at 50°C and 60°C for 3 h, respectively, 85% at 70°C and 80°C for 2 h and 1 h, respectively, 82% at 90°C and 100°C for 1 h, respectively, and 72% activity at 121°C for 40 min (Table 3). When the ultrafiltered concentrate was treated with surfactants, like SDS, Triton-X-100, tween 20, and urea, no inhibitory effect on the biological activity was observed. Also the biological activity remained the same at the exposure to *β*-mercaptoethanol and EDTA. However, with the treatment of TCA, a complete loss of activity was observed. In another case, 100% biological activity was found to be retained after treating the concentrate with 8 different metal salts. Contrary to this 70–90% activity was detected when antimicrobial substance was treated with various organic solvents (Table 3).

### 3.4. Partial Purification

**Figure 3:**
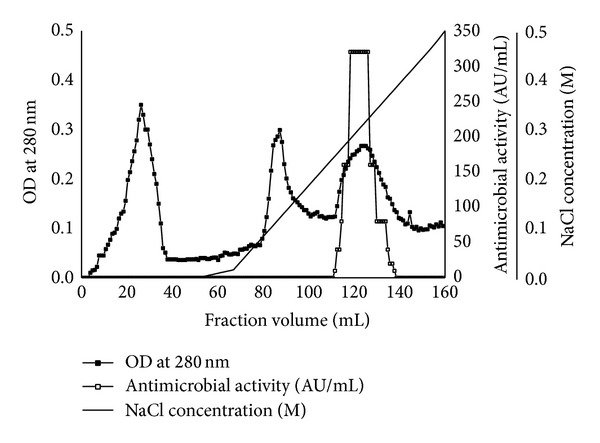
DEAE-Sepharose elution profile of antimicrobial peptide purification. Elution was performed with linear gradient of 0–0.5 M NaCl. Antimicrobial activity of all fractions was tested using* S. pyogenes* MTCC 442.

During the third step of purification, the dialyzate of 30% ammonium sulphate saturated fraction was loaded onto a DEAE-Sepharose anion exchange column and fractionated using a gradient of sodium chloride elution (0 to 0.5 M NaCl) wherein the fractions eluted between 0.25 and 0.5 M of NaCl, showed maximum activity (Figure 3). Among the three major peaks, the third peak showed antimicrobial activity against* S. pyogenes* MTCC 442. The active fractions were pooled together, desalted using n-butanol solvent and after complete removal of butanol in a speed vac (Martin Christe), protein profile was checked using SDS-PAGE (15%). The purification procedure, summarized in Table 4, resulted in approximately a total of 6400 U with 179-fold overall purification with a 26% recovery or yield of antibacterial activity. The N-terminal amino acid sequence was partially determined to be T-P-P-Q-S-X-L-X-X-G by a protein sequencer (model 494, Applied Biosystems, USA) in which X represents the blocked and unidentified amino acid. This N-terminal amino acid sequence was different from those of other antimicrobial peptides as reported from* Bacillus*.

### 3.5. Characterization of Antimicrobial Compound

**Figure 4:**
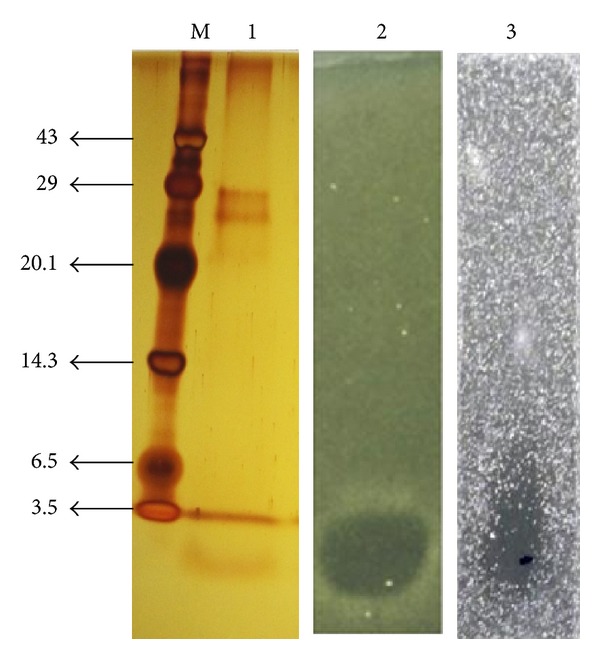
Tricine SDS-PAGE (15%) of antimicrobial compound from RLID 12.1, lane M (from left): low molecular weight marker, lane 1: partially purified compound after DEAE-Sepharose chromatography and lanes 2 and 3: zone of inhibition (zymogram) against* S. pyogenes* MTCC 1928 and 442.

The molecular weight of the band in Tricine-SDS-PAGE showing the antibacterial activity in zymogram, the band that shows clear zone of inhibition (blackish) against* S. pyogenes* (Figure 4), was estimated to be less than 2.5 kDa. TLC analysis revealed a spot showing negative result for both ninhydrin and lipid moiety but a white spot of *R*_*f*_ value 0.75 was observed when sprayed with water (photograph not shown).

### 3.6. Detection of* iturin C, fen D,* and* bmy B* Genes

**Figure 5:**
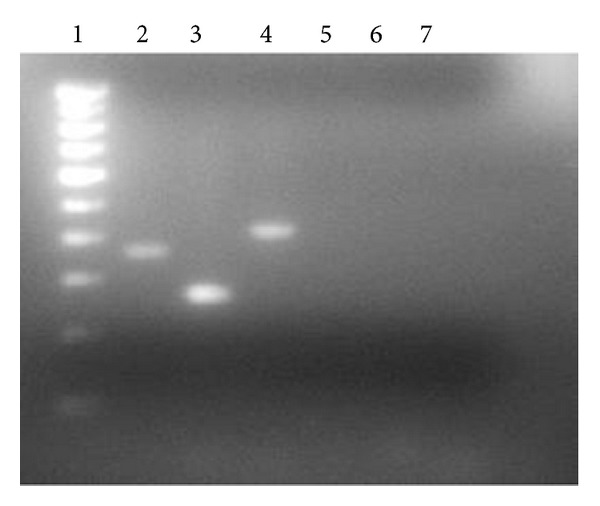
Lane 1: 100 bp DNA ladder, lanes 2, 3, and 4 show the PCR products formed using primers BMYB, FEND, and ITUC. Lanes 5–7 show negative controls without any bands.

This soil isolate, which displayed antagonistic activity, possesses the* iturin* C,* fengycin synthetase,* and* bacillomycin* genes, as evident from the amplification of these genes to PCR products. Agarose gel electrophoresis revealed that PCR products of about 300 and 800 bp were amplified from the genomic DNA of* B. subtilis* RLID 12.1 with primers shown in tabular form. As seen in Figure 5, a single clear PCR product of approximately 400 bp was observed when ITUC primers were used and 250 and 400 bp bands were observed when FEND and BMYB primers were used, indicating the most likely presence of the iturin operon in the chromosome. All PCR amplified (Figure 5) fragments were subsequently sequenced using the respective primers that were used for amplification. Sequence comparisons showed 99%, 97%, and 98% similarity or homology, respectively, between the sequences obtained from the amplified fragments and* iturin* C,* fengycin synthetase,* and* bacillomycin* genes in the database from the known surfactin producers.

### 3.7. Minimum Inhibitory Concentration (MIC)

The minimum inhibitory concentration of dialysed concentrate after ammonium sulphate fractionation for gram-positive bacteria (*S. pyogenes*) was found to be 0.25–0.5 *μ*gml^−1^, for gram-negative bacteria (*E. coli* MTCC 723 and* P. aeruginosa*) 32.0 and 8.0 *μ*gml^−1^, respectively, and for yeasts (*C. albicans* and* C. neoformans*) as 32.0 and 16.0 *μ*gml^−1^ (Table 5). The lowest MIC measured was 0.25 *μ*gml^−1^ for* S. pyogenes* MTCC 442. Partially purified compound after DEAE-Sepharose anion exchange failed to show any activity against gram-negative bacteria and yeasts but showed antibacterial activity against some gram-positive bacteria in the range 0.39–25 *μ*g/mL (Table 5). The minimum bactericidal values (MBC) ranged from 3.25 to 25.0 *μ*g/mL (Table 5).

## 4. Discussion

The antimicrobial substance found to be inhibitory to a wide spectrum of bacteria and yeasts was reproducibly demonstrable to a high level of antagonistic potential. In this investigation, RLID 12.1 showed both antibacterial (against gram-positive and gram-negative) and antimycotic activity against most of the hospital acquired infections. Prior to this study, a broad inhibitory spectral property had been reported in several strains, for example,* B. subtilis* LFB 112 [15]. The growth kinetics and inhibitory action of* B. subtilis* RLID 12.1 clearly revealed that the antimicrobial activity was detected after a steady state of growth curve particularly from the early to late stationary phase with its decline in the very late stationary phase (Figure 2). Since the antimicrobial activity of the test strain could be detected in the midstationary phase reaching the maximum at the late exponential phase with an arbitrary units of approximately 80 AU/mL, followed by a significant decrease during prolonged incubation of 72 h (Figure 2), it may be suggested that sporulation had perhaps no effect in this case on the production of antimicrobial substance.

The pH values of the culture broth rising from the initial pH of 7.4 to 8.0 during its growth have confirmed that the inhibitory action was due to the antimicrobial compound and not due to production of organic acids. Similar findings were found in* Bacillus* sp. FAS_1_ where the antimicrobial compound was produced at alkaline pH [16]. The antimicrobial substance appeared to be amazingly heat-stable as no reduction in the inhibitory activity was observed even after being heated to 100°C for 60 min or 121°C for 40 min. Besides this, the antimicrobial activity of ultrafiltered concentrate adjusted over a wide range of pH values (3.0–10.0) showed no drastic reduction. The heat and pH stability property of antimicrobial peptides were also reported in case of pumilicin and laterosporulin by* B. pumilus* and* Brevibacillus* sp., respectively [17, 18]. Subtilosin A (molecular mass of 3399.7 Da), produced by* Bacillus* sp., showed bactericidal [19] activity against* Listeria monocytogenes*, clinical isolates of* Gardnerella vaginalis* and* S. agalactiae* [20] and also against gram-negative bacteria. Shelburne et al. [21] showed high stability under extreme temperature and pH stresses, with retention of full activity after an hour of heat-treatment at 100°C or in the pH range of 2.0–10.0 [22]. Our observation about the antimicrobial substance produced by RLID 12.1 is in agreement with the previous observation. In another study, the strain* B. amyloliquefaciens* LBM 5006 isolated from the Brazilian Atlantic forest produced a bacteriocin like inhibitory substance (BLIS) of interest because of its broad antibacterial spectrum, which included activity against* L. monocytogenes, Bacillus cereus, Serratia marcescens,* and* Pasteurella haemolytica* [22]. The BLIS activity was associated with a peptide of approximately 5 kDa that was stable over the pH range of 3.0–8.0, heat stable (80°C, 30 min), and sensitive to proteolytic enzymes. The apparent resemblance of the physicochemical stability of this low molecular weight antimicrobial substance with RLID 12.1 except the resistance to proteolytic enzymes and exceptional heat-resistance warrants that the RLID 12.1 antimicrobial compound may be designated as BLIS. However, further experimentation is required to corroborate this finding. The ultrafiltered retentate of RLID 12.1 was found to be completely resistant to trypsin and pronase E; however, the reduction of its bioactivity to 72% (partial loss by 28%) when treated with high concentration of proteinase K and 100% loss of activity recorded after TCA treatment confirmed its proteinaceous nature. Resistance to nonproteolytic enzymes like lipase and *α*-amylase indicates that no lipid or glucosidic moiety was responsible for its biological activity. Failure of proteolytic digestion might be due to the presence of unusual amino acids with D-conformation in the peptide structure [23] due to the presence of proline residues or cyclic N and/or C terminally blocked peptides [24] which are in consonance with data reported from many* Bacillus* spp. [8, 17]. Our observation on the heat-stability profile and resistance to enzymes was also supported by a highly heat-stable (100°C for 1 h) antibacterial peptide produced by a Brazilian oil isolate* Bacillus firmus* H_2_O-1 that was found to be resistant to proteases and was stable at alkaline pH [25]. Chemical treatments with surfactants, metal salts, and EDTA did not cause any adverse effect on biological activity.

However when TLC analysis was performed using ion exchange derived antimicrobial fractions, ninhydrin-negative result indicated that N-terminal might either be blocked or modified which may be due to cyclization or chemical modification quite conspicuous to lantibiotics [26]. The partial N-terminal amino acid sequence of the antimicrobial peptide did not collectively match, either entirely or partially, with a single peptide or protein encoded by the genus* Bacillus*. However, it (TPPQS..) exhibited insignificant similarity to histidine kinase from* B. macauensis*, and TPPQ matches with mycosubtilin synthase subunit A [*B. subtilis* subsp. 351* spizizenii* str. W23, sequence ID: ref|YP_003866245.1]. The  ..PPQS.. matches slightly with surfactin synthetase [*Bacillus stratosphericus*; sequence ID: ref|WP_007498219.1.].

The presence of AMP biosynthetic genes in the* Bacillus* isolate in concern was determined by PCR using the specific primers for these genes (*bmy* B,* fen* D, and* itu* C). The sizes of the amplified products (Figure 4) precisely match the sizes of the PCR product in a study conducted earlier [10, 27]. In several earlier studies, in several strains of* Bacillus*, the biocontrol of plant pathogens was linked to the presence of the AMP biosynthetic genes* bmy B*,* fen D*,* itu C*,* srfA*, and* srfB* [10, 27]. It was reported earlier that in the* Bacillus* strain like FZB42,* bmy B,* and* fen D* genes were present whereas in QST713, RGAF51, and UMAF6639,* bmy B, fen D,* and* itu C* were present as AMP gene markers [10]. The similar observation has been made in the isolate RLID 12.1 that claims to possess the structural genes, namely,* bmy B, fen D* and* iturin C*. In a comprehensive study on the biosynthesis of surfactin, it was stated that sufactin production is inducible by actively growing cells with a postexponential synthesis [28].

It is essential to determine the MIC values of the substances to evaluate the antimicrobial efficacy. MIC and minimum bactericidal concentration (MBC) values of partially purified compound for* S. pyogenes* MTCC 442 and MTCC 1928 were found to be similar. But in the case of* B. cereus* and* Enterococcus faecalis*, MBC values were higher as compared to MIC values. However, MIC and MBC values for* Staphylococcus epidermidis* ATCC 12228 were found to be very high (>200 *μ*g/mL). Bacillomycin F [29] which effectively inhibited* C. albicans* (MIC, 40 *μ*g/mL) and* C. tropicalis* (MIC, 40 *μ*gml^−1^) exhibited modest inhibition against* Micrococcus luteus* (MIC, 200 *μ*g/mL) having no inhibitory effect on other bacteria tested (e.g., MIC > 400 *μ*g/mL:* E. coli* K12,* Streptomyces albus* G., and* S. aureus*). Likewise in our investigation, narrow-spectrum activity against gram-positive bacteria like* S. pyogenes, S. epidermidis, B. cereus,* and* E. faecalis* were found in partially purified antimicrobial compound.

However, no antimicrobial activity was observed against gram-negative organisms and yeasts as well as some gram-positive bacteria like* M. luteus* and* S. aureus,* which is in partial agreement with other previous observations [17]. Loss of antimicrobial activity against gram-negative bacteria and yeasts during the purification process and differences in MIC values between dialyzed and partially purified substance indicates that more than one antimicrobial compound might have been produced by RLID 12.1. In the present study, only one compound was partially purified using ion exchange chromatography. Thus our results suggest that there may be a combined production of one or more antimicrobial compounds that enabled a demonstration of broad spectrum activity by the dialysed concentrate which was almost similar as reported by Baindara et al. [30].

## 5. Conclusions

The present investigation reveals the broad-spectrum antimicrobial potential of the wild-type isolate RLID 12.1 identified as* B. subtilis*. It produces highly heat stable substances when compared with other antimicrobial proteins produced by other species of* Bacillus* and* Lactobacillus*. The present characterization revealed interesting properties which justifies its potential application in the biological control of pathogenic strains. Further biochemical and molecular characterization after complete purification will be undertaken.

## Figures and Tables

**Table 1:** Antimicrobial activity by agar well diffusion method.

S. number	Indicator organism	Antimicrobial activity (zone of inhibition in mm)
1	*Pseudomonas aeruginosa* MTCC 741	17.33 ± 0.44
2	*P. aeruginosa* MTCC 2582	21.5 ± 0.28
3	*P. aeruginosa* MTCC 424	17.83 ± 0.17
4	*Pseudomonas* sp. (clinical isolate)	17.50 ± 0.29
5	*Klebsiella pneumoniae *	15 ± 0.29
6	*K. pneumoniae* MTCC 109	15.17 ± 0.44
7	*Salmonella infantis* MTCC 1167	0
8	*Escherichia coli* MTCC 723	24.66 ± 0.16
9	*E. coli* MTCC 729	21.33 ± 0.33
10	*Acinetobacter baumannii* MTCC 1425	22.68 ± 0.15
11	*Proteus vulgaris* MTCC 1771	29.5 ± 0.28∗∗
12	*Yersinia aldovae* (arctic isolate)	21.67 ± 0.33
13	*Staphylococcus aureus* MTCC 737	0
14	*S. aureus* MTCC 96	14.5 ± 0.27
15	*S. aureus* NCIM 5021	0
16	*Streptococcus pyogenes* MTCC 442	33.17 ± 0.17
17	*S. pyogenes* MTCC 1928	28.5 ± 0.28
18	*S. pyogenes* NCIM 2608	17.33 ± 0.16
19	*Staphylococcus epidermidis* ATCC 12228	15.17 ± 0.16
20	*Carnobacterium maltaromaticum* (arctic isolate)	14.5 ± 0.27
21	*Enterococcus faecalis* APR 210	30.5 ± 0.26
22	*C. albicans *DI (GenBank accession number KJ095700)	14.33 ± 0.23
23	*C. albicans* WT (clinical isolate)	16.83 ± 0.44
24	*C. albicans* MTCC 227	13.67 ± 0.33
25	*C. albicans* MTCC 3471	0
26	*C. albicans* MTCC 854	17.33 ± 0.33
27	*C. albicans* SC 5314	12.34 ± 0.22
28	*C. glabrata* NCIM 3019	20.5 ± 0.29
29	*C. glabrata* MTCC 3814	16.83 ± 0.44
30	*C. krusei* NCIM 3129	23.33 ± 0.33
31	*Cryptococcus neoformans* NCIM 3541	24.33 ± 0.67
32	*Cryptococcus neoformans* NCIM 3378	15.33 ± 0.16

∗∗indicates cidal and static activity.

**Table 2:** Details of AMP biosynthesis gene primers for PCR amplification.

Name and sequences	Annealing	Reference
of primers used (5′ → 3′)	Temperature (°C)	
(1) ITURIN C	51	[10]
GAT GCG ATC TCC TTG GAT GT		
ATC GTC ATG TGC TGC TTG AG		
(2) FEN D	52	
GGC TGC TGC AGA TGC TTT AT		
TCG CAG ATA ATC GCA GTG		
AG		
(3) BMY B	50	
GAA TCC CGT TGT TCT CCA AA		
GCG GGT ATT GAA TGC TTG TT		

**Table 3:** Preliminary characterization of the cell-free supernatant from RLID 12.1.

Treatment	Concentration	Activity
Enzymes		
Control		+
Proteinase K	10 mg/mL	+
Pronase	10 mg/mL	+
Trypsin	10 mg/mL	+
Lipase	10 mg/mL	+
α-Amylase	10 mg/mL	+
pH		
3.0–10.0		+
Temperature		
5 hrs at 37°C		+
3 hrs at 50°C		+
3 hrs at 60°C		+
2 hrs at 70°C		+
1 hr at 80°C		+
1 hr at 90°C		+
1 hr at 100°C		+
40 mins at 121°C		+
Surfactants	1% (v/v)	+
EDTA	2, 5, and 50 mg/mL	+
TCA	10 mg/mL	−
Metals	1 mg/mL	+
Organic solvents	50% (v/v)	+

**Table 4:** Summary of purification steps.

Purification steps	Total Protein (mg)	Total Activity (AU)	Specific activity^a^	Purification fold	Yield, %
Cell-free supernatant (CFS)	13440	24000	1.785	1	100
Ultrafiltration	1000	16000	16	8.9	66.7
Ammonium sulphate precipitation (30%)	80.1	9600	119.85	35.85	40
DEAE-Sepharose chromatography	20	6400	320	179.27	26

^a^Specific activity is the ratio of total activity to that of total protein.

**Table 5:** MIC and MBC of partially purified antimicrobial compound.

S. number	Organisms	MIC of dialyzed concentrate (*μ*g/mL)	MIC of partially purified compound (*μ*g/mL)	MBC of partially purified compound (*μ*g/mL)
	Gram-negative bacteria			
1	*P. aeruginosa* MTCC 424	8.0	NA	Nil
2	*E. coli* MTCC 723	32.0	NA	Nil
	Gram-positive bacteria			
3	*S. pyogenes* MTCC 442	0.5	6.25	6.25
4	*S. pyogenes* MTCC 1928	0.25	3.125	3.125
5	*S. pyogenes* NCIM 2608	ND	NA	NA
6	*Bacillus cereus *	ND	0.39	3.125
7	*Enterococcus faecalis *	ND	25.0	25.0
8	*Staphylococcus epidermidis* ATCC 12228	ND	>200	>200
	Yeasts			
9	*C. albicans* MTCC 183	32.0	NA	Nil
10	*Cryptococcus neoformans* NCIM 3541	16.0	NA	Nil

NA: no activity; ND: not determined.
